# Can insulin signaling pathways be targeted to transport Aβ out of the brain?

**DOI:** 10.3389/fnagi.2015.00114

**Published:** 2015-06-16

**Authors:** Milene Vandal, Philippe Bourassa, Frédéric Calon

**Affiliations:** ^1^Faculté de Pharmacie, Université LavalQuebec, QC, Canada; ^2^Axe Neurosciences, Centre de Recherche du Centre Hospitalier de l’Université Laval (CHUL)Québec, QC, Canada; ^3^Institut des Nutraceutiques et des Aliments Fonctionnels, Université LavalQuébec, QC, Canada

**Keywords:** insulin, blood-brain barrier, amyloid beta-peptides, Alzheimer’s disease, brain capillary endothelial cells, insulin therapy, insulin transporter

## Abstract

Although the causal role of Amyloid-β (Aβ) in Alzheimer’s disease (AD) is unclear, it is still reasonable to expect that lowering concentrations of Aβ in the brain may decrease the risk of developing the neurocognitive symptoms of the disease. Brain capillary endothelial cells forming the blood-brain barrier (BBB) express transporters regulating the efflux of Aβ out of the cerebral tissue. Age-related BBB dysfunctions, that have been identified in AD patients, might impair Aβ clearance from the brain. Thus, targeting BBB outward transport systems has been suggested as a way to stimulate the clearance of Aβ from the brain. Recent data indicate that the increase in soluble brain Aβ and behavioral impairments in 3×Tg-AD mice generated by months of intake of a high-fat diet can be acutely reversed by the administration of a single dose of insulin. A concomitant increase in plasma Aβ suggests that clearance from the brain through the BBB is a likely mechanism for this rapid effect of insulin. Here, we review how BBB insulin response pathways could be stimulated to decrease brain Aβ concentrations and improve cognitive performance, at least on the short term.

## Introduction: Is There a Relationship Between Brain Aβ and the Symptoms of AD?

Similar to other neurodegenerative diseases, Alzheimer’s disease (AD) is a proteinopathy, in which accumulation of insoluble aggregates of Amyloid-β (Aβ), tau and TDP-43 occurs (Tremblay et al., 2007, 2011; Serrano-Pozo et al., 2011). Deposition of Aβ peptides into neuritic plaques is still instrumental in the neuropathological diagnosis of AD (Braak et al., 1993; Selkoe, 2001; Cummings, 2004), but whether it plays a causal role in the progressive dementia, which characterizes AD clinically, remains uncertain (Karran et al., 2011; Mormino, 2014; Morris et al., 2014). Nevertheless, one key element of information gathered in the last decade is the quantitative link between the deposition of Aβ42 and the risk of developing the disease.

Genetic data provide strong support for the role of Aβ in AD. Mutation or double copies leading to increased production lead to higher incidence of the disease. Indeed, mutations leading to overproduction all increase the risk of AD (Goate et al., 1991; Rogaev et al., 1995; Sherrington et al., 1995; Borchelt et al., 1996; Duff et al., 1996; Scheuner et al., 1996; Citron et al., 1997), while a mutation more recently found to reduce Aβ production was rather associated with reduced AD incidence (Jonsson et al., 2012). The discovery of mutations in amyloid precursor protein (APP) causing AD and the development of transgenic mouse lines overexpressing mutant APP reproducing many pathological features of AD all strongly suggest that accumulation of Aβ can cause AD, at least in its familial forms (St George-Hyslop, 2000; Bird, 2005; Gatz et al., 2006; Goedert and Spillantini, 2006; Roberson and Mucke, 2006; Haass and Selkoe, 2007). In fact, in the AD field, this simple quantitative link between Aβ and disease incidence is one of the rare postulates that remained true over the years. Facing a disease of intractable complexity, where lists of different key factors involved never stop growing, such a genotype-to-phenotype evidence offers a rare element of clarity. However, for sporadic AD, it should not be used for more than what it is, essentially a correlative link, and ultimately perhaps only a risk factor.

The role of Aβ in the symptoms of AD is truly one of the most controversial issues in the field. Most studies show significant correlation between insoluble Aβ or neuritic plaque and *ante mortem* cognitive symptoms (Blessed et al., 1968; Dickson, 1997; Tremblay et al., 2007). However, individuals with very high levels of Aβ do not necessarily develop cognitive symptoms (Knopman et al., 2003; Price et al., 2009; Rentz et al., 2010; Karran et al., 2011; Chételat et al., 2013) because other protective mechanisms are in play, including the so-called cognitive reserve (Rentz et al., 2010; Stern, 2012). Thus, it remains possible that Aβ is a consequence rather than a cause, or even a correlative event (Mormino, 2014; Morris and Tangney, 2014). There is indeed ground for a much more passive role of Aβ. For example, it has been argued that removal of Aβ plaques or tau tangles from the aged diseased brain could rather disrupt an ongoing compensatory mechanism and be harmful (Perry et al., 2000; Tayeb et al., 2012). The recent failure of a series of anti-amyloid treatments into late clinical trials only added more fuel to this counterargument (Golde et al., 2011; Tayeb et al., 2012; De Strooper and Chávez Gutiérrez, 2015). The hypothesis that clearing Aβ leads to improved cognition has only been confirmed in animal models (Mori et al., 2014; Pujadas et al., 2014; Vandal et al., 2014b). Although some evidence of effective clearing of Aβ exists after clinical trials, it is insufficient to state that any drug has succeeded in eliminating Aβ pathology (Selkoe, 2011; De Strooper and Chávez Gutiérrez, 2015). For example, active immunization trials with anti-Aβ antibodies have failed to reach primary clinical outcomes of improving cognition (Robinson et al., 2004; Grundman et al., 2013), despite reduced brain Aβ concentrations, possibly because of adverse vascular effects (Liu et al., 2012). A recent phase 3 clinical trial testing the γ-secretase inhibitor semagacestat, was stopped before the end of the trial because of major adverse effects such as infections, skin cancer and weight loss, which might explain the deterioration of cognitive function observed in treated patients (Doody et al., 2013; Desjardins et al., 2014). Notwithstanding the absence of cognitive benefit, a significant increase in CSF total Aβ42 was observed with semagacestat (Doody et al., 2013). Another limitation comes from the current imaging techniques that are used in clinical trials, which do not clearly detect the various pools or subforms of Aβ, such as soluble oligomers that are believed to be particularly toxic to synapses (Holland et al., 2014). Nonetheless, because of the strength of above-mentioned genetic evidence, the focus of therapeutic approaches in AD has thus long been on stopping Aβ overproduction, which may work at least in a subset of patients (Golde et al., 2011; Selkoe, 2011; Lane et al., 2012; Tayeb et al., 2012; Mullane and Williams, 2013). However, there is growing recognition that proteinopathies are more likely to stem from disequilibrium between production and clearance. Overproduction may not systematically lead to the disease if clearing mechanisms remain active. From a therapeutic perspective, decreasing the production of Aβ, using APP modulators or γ or β-secretase inhibitors, may be ineffective if clearance pathways are compensating. This hypothesis that neurodegenerative proteinopathies result from a ruptured equilibrium has been particularly applied to Aβ in AD, probably because of the impressive sum of available data deciphering its production and metabolism pathways. Whether such an equilibrium hypothesis sounds too simple for a slowly progressing disease remains to be established, but therapeutic strategies designed to enhance Aβ clearance from the brain have recently been considered amongst the most promising options to treat AD (Sagare et al., 2012; Wildsmith et al., 2013; Saito and Ihara, 2014).

## Aβ Clearance: the Role of the BBB

The blood-brain barrier (BBB) forms the major interface between the blood and brain tissues and can thus be considered as the gateway to the brain. The BBB is formed by brain capillary endothelial cells (BCEC) displaying a high metabolic activity and polarized expression of receptors and membrane transporters (Oldendorf, 1977; Cornford and Hyman, 2005; Weiss et al., 2009). Except for small lipophilic compounds, almost no molecule gets in or out of the brain, without some control exerted by the BBB. Accordingly, impressive amounts of data have underscored the role of the BBB in the regulation of Aβ concentrations in the brain. Using peripheral arterial and central venous blood samples, Bateman’s group has been able to determine venous to arterial (V/A) Aβ concentration ratios in non-demented patients (Bateman et al., 2006). Their data show that the V/A ratio of Aβ is increased in central venous samples, indicating that Aβ is continuously effluxed from the brain (Bateman et al., 2006). They further designed a calculation model integrating several parameters from their previous work (Potter et al., 2013) and estimated that 25% of total Aβ clearance from the CNS comes through direct transport across the BBB to the blood (Roberts et al., 2014).

BBB-expressed transporters such as the receptor for advanced glycation end products (RAGE) and low density lipoprotein receptor-related protein 1 (LRP1), are thought to play a key role in Aβ transport in and out of the brain (Deane et al., 2003; Kim et al., 2009). In addition, ATP-binding cassette (ABC) transporter family members, namely ABCB1, ABCG2, respectively known as P-glycoprotein and breast cancer resistance protein, and ABCG4, are also implicated in the efflux of Aβ, since pharmacological inhibition or gene deletion of these transporters can increase the brain uptake of Aβ (Cirrito et al., 2005; Kuhnke et al., 2007; Tai et al., 2009; Donkin et al., 2010; Do et al., 2012; Stukas et al., 2014). Altogether, these studies pinpoint the important role of BBB transporters in the efflux of Aβ and further suggest that their manipulation may be useful to alter brain Aβ concentrations.

## Evidence of BBB Dysfunction in Aging and in AD

Historically, the increased cerebrospinal fluid (CSF)/serum ratios of blood-borne macromolecules have been interpreted as an evidence of impaired BBB permeability in AD (Skoog et al., 1998). However, CSF concentration should be interpreted carefully as CSF presence is not a proof of BBB disruption, because circulating molecules have access to the CSF through the blood-CSF barrier at the choroid plexus (Pardridge, 2011; Strazielle and Ghersi-Egea, 2013).

An increase in the activity of RAGE (influx) vs. LRP1 (efflux) within the BBB has been suggested to contribute to the accumulation and deposition of Aβ in the brain (Donahue et al., 2006; Miller et al., 2008). Immunostaining experiments showed that RAGE expression is increased at the microvasculature of AD brains whereas LRP1 is decreased (Donahue et al., 2006; Miller et al., 2008). This increase in RAGE expression is associated with the progression of the disease (Miller et al., 2008), which supports evidence indicating that RAGE expression is enhanced in an Aβ-rich environment (Schmidt et al., 2000; Bierhaus et al., 2005). Both RAGE and LRP1 activities seem to be closely related since the blockade of RAGE-β interaction with a RAGE antibody enhances the expression of LRP1 in cultured human brain endothelial cells (Deane et al., 2004). This apparent disequilibrium between RAGE and LRP1 thus appears as an interesting therapeutic target for AD (Deane et al., 2009). Accordingly, the inhibition of RAGE as a therapeutic intervention expected to reduce Aβ influx and enhance its clearance, has been rapidly investigated. However, the phase 2 clinical trial provided disappointing results. AD patients randomized in the high-dose group displayed several adverse effects, including aggravated cognitive decline, whereas patients in the low-dose group displayed no significant cognitive improvement compared to the placebo group (Galasko et al., 2014).

Morphological abnormalities of brain capillaries, as well as evidence of cerebrovascular dysfunction such as decreased cerebral blood flow (CBF) or lower brain glucose uptake and metabolism, have also been documented in normal aging (Farrall and Wardlaw, 2009; Erickson and Banks, 2013; Nugent et al., 2014; Montagne et al., 2015) and AD patients (Wang et al., 2006; Taheri et al., 2011; Sagare et al., 2012; Erickson and Banks, 2013). Supportive observations have also been gathered in animal models of Aβ or tau AD-like neuropathology (Paul et al., 2007; Bourasset et al., 2009; Mehta et al., 2013; Do et al., 2014). More recently, apolipoprotein E4 (apoE4) has been associated with series of BBB defects, including impaired Aβ clearance (Deane et al., 2008; Salem et al., 2015), reduced glucose and polyunsaturated fatty acids uptake, decreased microvascularization and CBF (Reiman et al., 2004; Bell et al., 2012; Sagare et al., 2012; Vandal et al., 2014a; Alata et al., 2015). Surgical CBF reduction in an animal model of AD has been shown to initiate a vicious cycle between Aβ neuropathology and CBF deficits (Li et al., 2014). These evidence support data indicating that Aβ can also be cleared by perivascular drainage of the interstitial brain fluid (Preston et al., 2003; Xie et al., 2013), a clearance pathway thought to be driven by the CBF pulsation force (Schley et al., 2006; Weller et al., 2009), but losing its strength with age (Schley et al., 2006; Weller et al., 2009; Kress et al., 2014). Reduced CBF observed in aging and in AD patients may thus impair Aβ clearance potentiating its accumulation and aggregation in the CNS (Erickson and Banks, 2013; Desjardins et al., 2014; Li et al., 2014; Oudegeest-Sander et al., 2014), which can further damage the cells of the neurovascular unit. RAGE-Aβ complex may play a predominant role in the decrease of CBF in AD. Upon binding with Aβ, RAGE triggers the release of endothelin-1, a potent vasoconstrictor, and proinflammatory factors, which can indirectly reduce the CBF (Deane et al., 2003). Finally, patrolling monocytes have also been shown to adhere to cerebral microvessels triggering the internalization of vascular Aβ, and may be implicated in another clearance process thought to become defective in AD (Michaud et al., 2013). Altogether, these series of data provide strong arguments for a dysfunction of the BBB associated with AD.

## Insulin Signaling Pathway in the BBB

Insulin exerts a plethora of effects in the CNS. Centrally administered insulin increases glycemia and reduces blood insulin, effects that are often at the opposite of those in the periphery (Banks et al., 2012; Fernandez and Torres-Alemán, 2012). In addition to its role in the maintenance of energy balance, insulin is implicated in the regulation of autonomic outflow and neurotrophic factors (Banks et al., 2012; Fernandez and Torres-Alemán, 2012). Evidence of reduced cerebral perfusion has been found in insulin resistant or diabetic patients (Novak et al., 2006, 2011; Brundel et al., 2012; Rusinek et al., 2015). Although local synthesis has been evidenced (Plata-Salamán, 1991; Banks, 2004), most insulin action in the brain probably comes from circulating insulin (Margolis and Altszuler, 1967; Banks et al., 1997a; Banks, 2004). Insulin can be transported across the BBB by three mechanisms: extracellular pathway, saturable transmembrane diffusion and via the choroid plexus. Permeability of the BBB to insulin is variable among brain regions. It is estimated that insulin crosses the BBB 2–8 times faster in the olfactory bulb, the most insulin receptor (INSR)-enriched region, than in the whole brain (Banks et al., 1999). Nevertheless, to put that in perspective, less than 0.05% of intravenously injected insulin (per gram of whole brain) enters the mouse brain (Banks et al., 1997a, 2012; Banks and Kastin, 1998).

**Figure 1:**
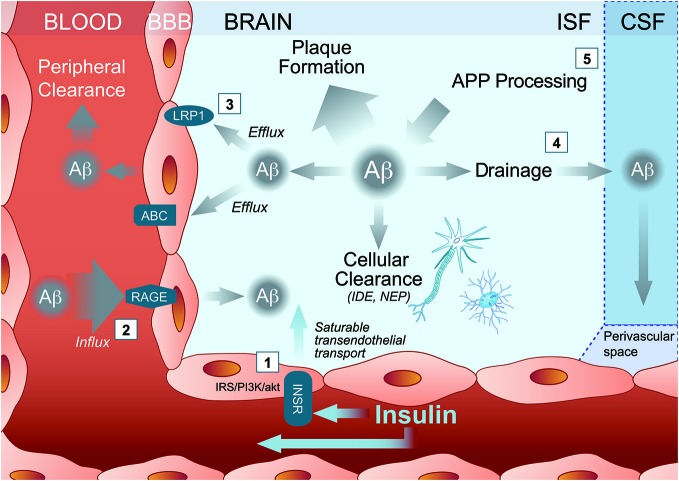
**Multiple pathways through which insulin may activate Amyloid-β (Aβ) clearance out of the Alzheimer’s disease (AD) brain. (1)** Peripherally administered insulin binds the insulin receptor (INSR) either to trigger cell-signaling pathways within brain capillary endothelial cells or to ferry an insulin molecule into the brain parenchyma through a saturable transendothelial transport mechanism. Insulin then might affect brain Aβ clearance by modulating **(2)** influx transporter such as receptor for advanced glycation end products (RAGE) or **(3)** efflux transporters such as ATP Binding Cassette transporter (ABC) and Low density lipoprotein receptor-related protein 1 (LRP1). **(4)** Insulin inside the brain may also increase Aβ drainage to the CSF and/or **(5)** reduce Aβ production. ABC, ATP Binding Cassette transporter; APP, amyloid precursor protein; Aβ, Amyloid-β; BBB, blood brain barrier; CSF, cerebrovascular fluid; IDE, insulin degrading enzyme; INSR, insulin receptor; IRS/PI3/Akt, insulin receptor substrate (IRS)/phosphoinositide-3 kinase (PI3)/Akt pathways; ISF, brain interstitial fluid; LRP1, Low density lipoprotein receptor-related protein 1; NEP, neprilysin; RAGE, receptor for advanced glycation end products.

Insulin is ferried into the brain via the INSR (Frank et al., 1985; Duffy and Pardridge, 1987; Banks et al., 1997a, 2012; Banks and Kastin, 1998) located at the luminal surface of BCEC (Miller et al., 1994; Figure 1). This transendothelial transport is saturable (King and Johnson, 1985; Hachiya et al., 1988), at a rate dependent on plasma insulin concentrations (Baura et al., 1993). First, insulin binds its receptor at the luminal side of the BBB and receptor-mediated endocytosis occurs. Next, the insulin and insulin-receptor complex are transported into the endothelial cytoplasm. Finally, insulin is exported out of the endothelial cell inside the brain parenchyma through receptor-mediated exocytosis (Pardridge, 1986).

In addition to the active transport insulin across the BBB, INSR also modulate several BCEC function through insulin intracellular signaling pathways (Banks et al., 2012; Figure 1). When insulin binds and activates INSR, it undergoes autophosphorylation, which induces the recruitment of insulin signaling proteins, such as insulin receptor substrate (IRS)-1 and activation of the phosphoinositide 3-kinase/protein kinase B (Akt) pathway (Fernandez and Torres-Alemán, 2012). Experiments in cultured BCEC confirm the induction of such a signaling pathway by insulin (Katakam et al., 2009). Since INSR are expressed by endothelial cells throughout the BBB network (Miller et al., 1994), the downstream action of insulin in the brain can thus be widespread in all the perfused cerebral tissue.

INSRs are widely distributed in the CNS. In mice, immunohistochemistry and *in situ* hybridization experiments showed that insulin receptors are mainly found in the olfactory bulb followed by the cerebral cortex, the hippocampus, the hypothalamus and the cerebellum (Havrankova et al., 1978; Unger et al., 1989; Fernandez and Torres-Alemán, 2012). Smaller amounts of INSR are also found in the striatum and the thalamus (Fernandez and Torres-Alemán, 2012). Insulin interacts with receptors on neuron and glial cells (Unger et al., 1989). In contrast to the periphery, glucose uptake in the CNS is independent of insulin (Hasselbalch et al., 1999). Interestingly, the role of insulin in the brain appears to be older from an evolutionary standpoint, more closely related to a growth factor, specifically, similar to the Insulin-like Growth Factor (IGF; Banks et al., 2012). Insulin signaling in the brain is linked to neuronal survival, synaptic and dendritic plasticity, learning, memory and formation of neuronal circuits (Banks et al., 2012; De Felice and Ferreira, 2014; Kleinridders et al., 2014). Therefore, although the physiological effects of insulin in cerebral tissues are very different than in periphery, there is not much reason to believe that its cellular signaling pathways within BBB endothelial cells are very different than in other tissue like muscles or liver. The main difference is that impact of insulin on parenchyma brain cells has first to go through the BBB, whether by actual transport or via cell signaling, under the tight regulation of BCEC of the capillary network.

## Can Insulin Trigger Aβ Efflux Though the BBB?

The benefits of intranasal insulin in AD patients led to the hypothesis that insulin might be a therapeutic tool in AD (Craft et al., 2012; Freiherr et al., 2013; Claxton et al., 2015). In parallel, studies have been conducted in mouse models of AD to elucidate the mechanisms by which insulin might modulate AD-like neuropathology and cognition. Consistent with evidence showing that nutritional factors alter AD risk (Morris, 2009; Hennebelle et al., 2014; Morris and Tangney, 2014), high-fat-diets (HFD) have been consistently shown to further increase Aβ concentrations in the brain of APP models (Ho et al., 2004; Maesako et al., 2013; Ramos-Rodriguez et al., 2014), as well as in the 3×Tg-AD model (Julien et al., 2010; Barron et al., 2013; Vandal et al., 2014b). Thus, the combination of HFD with APP overexpression generates a model of diabetic AD mice, useful to investigate the links between T2D and AD observed in humans.

In 3×Tg-AD mice fed with the HFD for 9 months, we have recently shown that an acute insulin injection (3.8 U/kg body weight) corresponding to approximately 2000-fold the normal fasting insulin level in the mouse, restores cortical soluble Aβ40 and Aβ42 back to the level of mice fed with the control diet (CD). The Aβ lowering affect of insulin was accompanied with improved memory function in HFD-fed 3×Tg-AD mice (Vandal et al., 2014b). We also identified changes in molecular markers implicated in Aβ production, all altered by a single insulin injection, including increased α-APP, increased X11α, decreased BACE, and decreased autophagy-related proteins (Kamenetz et al., 2003; Kondo et al., 2010; Son et al., 2012). Although these observations suggest that APP/Aβ production may be affected by insulin, as previously shown (Pandini et al., 2013; Wang et al., 2014), the rapidity of insulin effect suggests that other mechanisms are in play. Indeed, previous evaluation of the synthesis and turnover of Aβ in an APP mouse model led to an estimation of a half-life ranging from 1.0–2.5 h for Aβ, C99 and APP, respectively (Savage et al., 1998). In line with this observation and with findings from previous investigators (Cirrito et al., 2003; Barten et al., 2005; Abramowski et al., 2008), Basak et al. used ^13^C_6_-leucine injection and Liquid Chromatography/Mass Spectrometry (LC/MS) analysis to evaluate clearance of Aβ from the brain of APP mice and found an Aβ half life ranging from 2.8–2.9 h (Basak et al., 2012). Indeed, a slow brain clearance rate for Aβ (18–33 h) was reported in monkey, using the same methodology based on ^13^C_6_-leucine injection. As the metabolic rate in humans is slower than in the mouse (Potter et al., 2013), Aβ half-life is likely to be shorter in the mouse as well. Therefore, the turnover rates of Aβ in the brain suggest that the insulin-induced downregulation effect of soluble Aβ is unlikely to be solely explained by changes in production.

On the other hand, BBB clearance mechanisms could occur faster. In our experiments in HFD-fed 3×Tg-AD mice, we indeed observed a concomitant increase of plasma Aβ42 following insulin injection strongly suggesting that insulin increased Aβ clearance from the brain (Vandal et al., 2014b). This hypothesis is in line with human studies reporting an increase of plasma Aβ following insulin administration (Kulstad et al., 2006; Karczewska-Kupczewska et al., 2013). The cerebrovasculature is so dense throughout the brain that it is conceivable that the network of capillaries, venules and veins can excrete rapidly Aβ out of the brain.

### How can Insulin Modulate Aβ Clearance from the Brain?

A first obvious mechanism of insulin effect on Aβ clearance is through the activation/inactivation of Aβ transporters at the BBB. Binding of insulin to its receptor at the BBB and transport across the BBB can occur rapidly after IV insulin administration (Figure 1). Since INSR is widely distributed in the brain, insulin can target several cerebral regions (Banks et al., 2012). Interestingly, the hippocampus, a brain region accumulating large amounts of fribrillar Aβ during AD progression, is also highly enriched in INSR (Mirra et al., 1991; Kadir et al., 2011; Banks et al., 2012). Studies in mice revealed that intravenously administered radiolabelled-insulin can be detected in mouse brain 1 min after injection (Banks et al., 1997a,b, 1999; Banks and Kastin, 1998; Yu et al., 2006). In a time frame of 20 min following IV administration, insulin modulates the BBB transport and analgesic effect of an opioid receptor agonist in mice suggesting that central effect of insulin appears minutes following peripheral administration (Witt et al., 2000).

Insulin has already been shown to potentiate the brain transport of molecules such as leptin (Kastin and Akerstrom, 2001) and amino acids (Tagliamonte et al., 1976). In streptozotocin-treated mice, insulin increased cerebral microvessels expression of occludin, claudin-5, and ZO-1 (Sun et al., 2015). Although little evidence is available regarding the effect of insulin on the expression of LRP1 at the BBB, insulin regulates translocation and uptake of LRP1 receptor in hepatic cells (Laatsch et al., 2009). Interestingly, LRP1 expression is downregulated in brain capillaries of streptozotocin-injected mice (Hong et al., 2009) and CSF soluble LRP1 is increased in type 1 diabetes patients treated with insulin for several years (Ouwens et al., 2014), suggesting that insulin might increase central LRP1, at least on the long term. Finally, insulin might also modulate RAGE concentration. Soluble RAGE is inversely correlated with plasma insulin concentration during an oral glucose tolerance test in healthy human subjects (Forbes et al., 2014). In isolated brain microvessels from streptozotocin-injected mice, insulin reduces the concentration of RAGE compared to diabetic mice (Sun et al., 2015). On the other hand, there is a wealth of evidence showing that insulin modulates the levels and function of ABC transporters such as P-glycoprotein in cultured BCEC (Liu and Liu, 2014). Although no data actually confirms that insulin modulate the activity LRP1, RAGE or other BBB Aβ transporters within the time frame of minutes, such an action simultaneously throughout the dense brain capillary network could in principle underlie the rapid effect observed with insulin on Aβ efflux (Figure 1).

Other more speculative mechanisms of insulin include enzymatic degradation of Aβ after insulin administration. However, no changes in insulin-degrading enzyme levels were detected, arguing against a direct effect of insulin on these enzymes known to catalyze the degradation of Aβ present in brain microvessels (Miller et al., 1994; Banks et al., 2012). On the other hand, drainage of Aβ from central interstitial fluid to CSF is increasingly recognized as a key clearance mechanism of brain Aβ. Experiments with intraparenchymal radiotracer injections show that significant amount of Aβ is effluxed through paravascular glymphatic system from the interstitial space to the CSF (Kress et al., 2014). This view is also supported by clinical evidence: low concentrations of CSF Aβ is now considered as a diagnostic tool for AD (Palmqvist et al., 2014). Therefore, insulin could also lead to a drop in brain soluble Aβ through interaction in water channels or by increasing CSF turnover (Zemva and Schubert, 2014). In support of this view, the administration of intranasal (Grichisch et al., 2012; Schilling et al., 2014) or intravenous (Kerr et al., 1993; Tallroth et al., 1993; Powers et al., 1996; Kennan et al., 2005) insulin have been linked to an increased CBF in human. Consistent effects of insulin on blood flow dynamics have been reported in mice after a stroke (Tennant and Brown, 2013). Furthermore, systemically administered insulin increases in CSF Aβ42 levels, particularly in the subjects with improved memory (Watson et al., 2003). The cerebrovascular response to insulin appears to be biphasic: first a vasoconstriction at low doses, then a vasodilation at higher doses, accompanied by an inhibition of nitric oxide synthase (Katakam et al., 2009). Therefore, since the paravascular drainage may be enhanced in parallel with CBF (Schley et al., 2006; Weller et al., 2009), modulation of CBF could also contribute to insulin-induced Aβ clearance. However, studies on the effect of brain-CSF transport led to apparent contradictory data. Intracerebral co-injection of very high doses of insulin with ionidated Aβ40 into the parietal cortex of rats led to less diffusion of radioactivity in the CSF (Shiiki et al., 2004), suggesting that systemic insulin has a different impact on Aβ than CNS insulin. This also argues for a direct effect of insulin on luminally exposed BBB transporters and CSF Aβ in human subject following insulin infusion (Figure 1).

### Alternative Therapeutic Strategies

Because of the well-known side effects resulting from the long-term use of insulin, authors have tried to use intranasal administration in clinical trials with AD patients (Freiherr et al., 2013). Importantly, intranasal insulin improved memory function in patients suffering from mild cognitive impairment and AD (Reger et al., 2006, 2008; Craft et al., 2012). Despite the fact that changes in peripheral glucose metabolism are observed following intranasal insulin administration (Dash et al., 2015; Gancheva et al., 2015), no significant change in blood insulin were detected (Born et al., 2002; Hallschmid et al., 2008). However, if peripheral mechanisms contribute to insulin-induced central Aβ clearance (Zhang and Lee, 2011), intranasal administration might then be less effective than direct peripheral administration.

Another concern linked to insulin administration is that the effect of insulin might not be sustainable on the long-term due to desensitization or other compensatory mechanisms. Indeed, INSR down-regulation and desensitization have been described in insulin resistance, including in cerebral tissues (Ketterer et al., 2011). As brain insulin resistance is likely to be present in most AD patients (Steen et al., 2005; Fernandez and Torres-Alemán, 2012; Talbot et al., 2012), the effect of chronic treatment with insulin on Aβ clearance might wear-off over time. Various INSR agonists could be useful in such a case. For example, partial agonist (or even antagonist) could be used to exert a chronic impact on these clearance mechanisms without inducing tolerance. Insulin analogs such as insulin detemir and insulin glargine are widely used to treat diabetic patients and are very effective to lower fasting glucose (Pollock et al., 2011). Nonetheless, those analogs have the disadvantage, when administered in the periphery, of inducing hypoglycemia and weight gain, which is associated to a higher risk of cardiovascular disease (Niswender, 2011). Xmet is a high affinity allosteric human monoclonal antibody that targets the INSR. When binding the INSR, Xmet mimics the glucoregulatory but not the mitogenic effect of insulin. In an animal model of diabetes, Xmet normalizes glucose tolerance without weight gain and hypoglycemia (Bhaskar et al., 2012). Although the central effect of Xmet and its capacity to cross the BBB still have to be determined, Xmet might represent an interesting therapeutic tool in AD as well.

Although insulin is an interesting therapeutical tool in AD, several parameters remain to be considered. First, in our previous study, we have shown that transgenic mice are glucose intolerant and that the glucose intolerance progressed to decreased insulin sensitivity and reduction in insulin production when the mice were fed a HFD (Vandal et al., 2014b). This raises the question whether insulin resistance and basal insulin levels affect the Aβ clearance capacity of insulin. Indeed, it is estimated that 46% of AD patient have impaired fasting glucose (Janson et al., 2004) and data suggest that a majority of AD patients have central insulin resistance (Steen et al., 2005; Talbot et al., 2012), suggesting that impaired insulin signaling might be a part of AD pathological process. Consequently, the possible impact of insulin resistance on the Aβ clearance capacity of insulin has to be taken into account in future studies.

## Conclusion

Although perhaps too simple, the facilitation of Aβ clearance out of the brain represents a conceptually attracting therapeutic strategy to reduce Aβ burden in cerebral tissue. A game-changer has been the quantitative kinetic work of Bateman and colleagues indicating that it is possible to measure with relative accuracy Aβ clearance, even sufficiently to be used as surrogate marker in clinical assays. It is unclear whether a disease modifying or simply a symptomatic effect can be expected, but further preclinical and clinical studies appear to be worthwhile. The timing of the intervention is probably important as the stimulation/potentiation of Aβ clearance is likely to be more useful to prevent Aβ accumulation, whether as primary or secondary prevention measures, rather than when dementia signs have become obvious, and therefore when it might be too late. Thus, the control of Aβ clearance might be part of future risk management perspectives, similar to the treatment of cardiovascular diseases, in the hope of decreasing the likelihood of developing AD. However, to achieve this, better understanding of BBB clearance mechanisms are still needed. In this regard, the recent evidence covered in this review suggest that insulin through its transport into the brain or its signaling pathways within cerebral endothelial cells offers promising opportunities to increase Aβ clearance.

## Conflict of Interest Statement

The authors declare that the research was conducted in the absence of any commercial or financial relationships that could be construed as a potential conflict of interest.
